# Homology Modeling of the CheW Coupling Protein of the Chemotaxis Signaling Complex

**DOI:** 10.1371/journal.pone.0070705

**Published:** 2013-08-07

**Authors:** Derek J. Cashman, Davi R. Ortega, Igor B. Zhulin, Jerome Baudry

**Affiliations:** 1 Department of Biochemistry and Cellular and Molecular Biology, University of Tennessee, Knoxville, Tennessee, United States of America; 2 UT/ORNL Center for Molecular Biophysics, University of Tennessee, Knoxville, Tennessee, United States of America; 3 Department of Physics, University of Tennessee, Knoxville, Tennessee, United States of America; 4 Joint Institute for Computational Sciences, Oak Ridge National Laboratory, Oak Ridge, Tennessee, United States of America; 5 Department of Microbiology, University of Tennessee, Knoxville, Tennessee, United States of America; German Research School for Simulation Science, Germany

## Abstract

Homology models of the *E. coli* and *T. maritima* chemotaxis protein CheW were constructed to assess the quality of structural predictions and their applicability in chemotaxis research: i) a model of *E. coli* CheW was constructed using the *T. maritima* CheW NMR structure as a template, and ii) a model of *T. maritima* CheW was constructed using the *E. coli* CheW NMR structure as a template. The conformational space accessible to the homology models and to the NMR structures was investigated using molecular dynamics and Monte Carlo simulations. The results show that even though static homology models of CheW may be partially structurally different from their corresponding experimentally determined structures, the conformational space they can access through their dynamic variations can be similar, for specific regions of the protein, to that of the experimental NMR structures. When CheW homology models are allowed to explore their local accessible conformational space, modeling can provide a rational path to predicting CheW interactions with the MCP and CheA proteins of the chemotaxis complex. Homology models of CheW (and potentially, of other chemotaxis proteins) should be seen as snapshots of an otherwise larger ensemble of accessible conformational space.

## Introduction

Bacterial chemotaxis is widely used as a model to study signal transduction in biological systems. The core signaling complex in chemotaxis consists of chemoreceptors and the histidine kinase, CheA, that are linked by the coupling protein, CheW. Chemoreceptors detect various extracellular and intracellular stimuli and modulate CheA activity, which transduces the signals to the flagellar apparatus via its cognate response regulator, CheY [1], [2]. In many organisms, the signaling complex assembles into organized arrays at the cell poles, where chemoreceptors cooperatively regulate kinase activity [3], [4]. This high-order structure is critical for signal amplification, the remarkable sensitivity of the system, and its precise adaptation [5], [6]. Although the general concepts involved in the chemotaxis pathway are understood, the details of the molecular mechanisms are still a matter of intensive research [7], [8] and an atomic description and complete molecular analysis of the chemotaxis components is fundamental to address this challenging topic [9].

Indeed, studying a large, multi-protein complex requires the use of more than one structural biology technique. The complex is too large to be studied with a single X-ray crystal structure or NMR ensemble. Cryo-electron tomography has been used recently to obtain low resolution structures of this complex. The electron density maps were used for low-resolution “docking” of previously experimentally obtained X-ray and/or NMR structures, providing an overview of the complex in its entirety [10], [11]. Additional approaches, such as computational modeling, have been used to explore such low-resolution complex models [12]. While coarse-grained modeling techniques can be used to understand the arrangement of basic elements of the complex, atomic resolution is necessary for understanding the molecular mechanism of signal transduction.

However, the structural knowledge of the chemotaxis signaling complex at the atomic level is incomplete and available mostly for two model organisms: *Thermotoga maritima*, a model organism for protein crystallography, and *Escherichia coli*, a model organism for chemotaxis [13], [14]. Other model organisms for chemotaxis, such as *Rhodobacter sphaeroides* and *Bacillus subtilis* [15], [16], still do not have resolved three-dimensional structures available for their chemotaxis signaling complexes. This is in sharp contrast with the large quantity of sequences known for chemotaxis proteins [17]; there are 3,738 CheW protein sequences from draft and complete genomes in the Microbial Signal Transduction (MiST2) database as of August 2012 [18]. To translate this wealth of sequence data into structural knowledge, it becomes necessary to use *in silico* approaches to build molecular models. For example, homology models of CheW proteins from the human pathogen *Borrelia burgdorferi* have been built recently using the NMR structure of *T. maritima* CheW as a template [19].

Homology modeling has been used extensively in a wide variety of applications, including analyzing ligand binding sites [20], [21], substrate specificity [22], docking and scoring involved in rational drug design [23], generating ensembles for docking [24], generating and analyzing binding sites for protein-protein interactions [25], as well as providing starting models in X-ray crystallography [26] and NMR spectroscopy [27]. In homology modeling, the higher the sequence identity between the protein sequence to be modeled (the target), and the protein template, the higher the quality of the model [28]. Sequence identity levels of less than ∼30% between the template and the target proteins often results in poor quality models. Thus, proteins in this range of sequence identity are often referred to as being in the “Twilight Zone” of homology modeling [29]. This is the case for CheW, where functional homologs may exhibit very low sequence identity [30]. CheW also shares a similar fold with the P5 domain of the CheA kinase, while also having low sequence identity with this domain. This is particularly interesting because these two protein structures have been proposed to bind to each other and interact in a similar fashion [10], [11].

It is therefore very important for current and future structural studies to quantify the level of confidence one can have in homology models of CheW, a protein with the lowest sequence identity among components of the signaling complex. The present work addresses this question by assessing the quality of structural predictions and the extent to which they can explain and rationalize the function of the corresponding proteins.

## Materials and Methods

### Bioinformatics

CheW sequences were retrieved from complete genomes in the August 2012 release of the MiST2 database [18]. The sequences were then pruned using the CheW domain definition from the Pfam [31] model PF01584 with the HMMER3 software [32] and 2,240 sequences with a single hit to the Pfam model were selected. A multiple sequence alignment was generated using *linsi* from the MAFFT package [33]. Sequences with 98% identity were deleted to avoid redundancy. The final dataset contained 1,742 sequences that were re-aligned.

### Homology Modeling

For the modeling of *E. coli* CheW, the sequence was obtained from the UniProt database (Entry ID: **P0A964**) [34], and modeled based on the *T. maritima* CheW structure as the template obtained from the Protein Data Bank [35] (PDB ID: **1K0S**) [14]. Similarly, the *T. maritima* CheW protein was modeled from its sequence (UniProt Entry ID: **Q56311**) using the *E. coli* CheW structure as the template (PDB ID: **2HO9**) [15]. The first model in the NMR structures was used as a template for the homology modeling. The program MOE, version 2010 (Chemical Computing Group, Inc., Montreal, Quebec, Canada), was used to align the sequences of CheW for *E. coli* and *T. maritima* against each other using the BLOSUM62 substitution matrix, with a gap start penalty value of 7 and a gap extension penalty value of 1. This sequence alignment is very close to that obtained from the multiple sequence alignment as shown in Supporting Information (Figure S1 in File S1), which reveals that multiple sequence alignment-based and pairwise *E. coli* vs. *T. maritima* CheW alignments produce nearly identical results. Twenty homology models (i.e. the same number of models that in the NMR structures) were built for both the *E. coli* and *T. maritima* CheW proteins. The C-terminal and N-terminal outgap modeling and automatic disulfide bond detection options were enabled in MOE. The models generated were scored based on Coulomb and Generalized Born interaction energies [36], and the top scoring homology model was selected for molecular dynamics and Monte-Carlo simulations.

### Molecular Dynamics Simulations

The dynamics of the selected CheW homology models was investigated using all-atom molecular dynamics (MD) simulations, including the top scoring homology model generated for *E. coli* and *T. maritima* CheW. In addition, the dynamics of the first NMR structure (which is also the most thermodynamically favorable) of the PDB entries for the corresponding proteins (1K0S and 2HO9) was also simulated. Each protein was solvated using periodic boundary conditions with 8,067 TIP3P water molecules [37], [38]. The molecular dynamics program NAMD2 version 2.7 [38] was used with the CHARMM22 all-atom force field at a simulated temperature of 300 K. The integration step was set to 2 fs and all of the distances in the system involving hydrogen atoms were constrained to equilibrium values. All simulated systems were initially energy minimized using the conjugate gradient algorithm for 2,000 steps. After initial energy minimization, the systems were gradually heated in an equilibration procedure from 100 K to 300 K, in incremental steps of 50 K for 100 ps at a time, for a total of 500 ps. This was followed by a production run of 50 ns (Supplementary Information, Figure S2 in File S1).

### Monte Carlo Simulations

The same systems that were used in the MD simulations were used in Monte-Carlo simulations using the LBMC method [39]. All simulations were run using an equilibration phase of 3×10^8^ Monte Carlo MC steps, followed by a total of 3×10^9^ MC steps (Supplementary Information; Figure S2 in File S1). The simulation temperature was chosen to be slightly below the unfolding temperature, based on 13 short simulations of 3×10^8^ MC steps, i.e. of k_B_T/ε = 0.7, where ε is the depth of the Gō potential [40], [41]. Frames were saved every 10^5^ MC steps. Trial moves consisted of swapping three consecutive peptide planes per step and/or changing the corresponding *Ψ* angles [39] with an acceptance rate of approximately 20–25%; setting the fraction of local moves to 10% and the fraction of *Ψ*-only moves to 30%. RMSDs were calculated using the same numbers of residues as with the MD simulations (above), and for the LBMC ensembles, 33,000 structures were generated in the molecular dynamics simulations of the selected homology model and NMR model.

### Sequence and Structural Similarities

Sequence similarity was measured and mapped to the *E. coli* NMR structure with the MultiSeq tool [42] of the VMD package [43] using the BLOSUM60 similarity matrix. The RMSD (root-mean-square deviation) per residue between two structures as a measure of structural similarity were calculated using a custom script for VMD and included all heavy atoms for each residue. The overall similarities and differences between two given structures were quantified by calculating RMSD between these structures. Three different RMSDs were calculated that focus on different structural subsets of CheW: i) using the backbone heavy atoms of all residues, ii) using the backbone heavy atoms of only the “protein core”, i.e. excluding residues 1–16 and 158–167 in the *E. coli* protein, or residues 1–9 and 148–151 in the *T. maritima* protein, and iii) using only the backbone heavy atoms of only the α/β consensus residues, i.e.; residues having either a α-helix or β-pleated sheet structure in all 20 sub-structures of the NMR models. For the *E. coli* protein, this included residues 17–19, 22–24, 27–30, 36–39, 57–61, 64–69, 87–93, 96–102, 104–105, 109–111, 133–135, 142–144, and 154–160 (57 residues, or 34.1%, out of the total 167 residues in the protein). For the *T. maritima* protein, the residues included were 12–17, 22–26, 30–34, 51–55, 58–63, 65–69, 80–84, 92–95, 97–103, 127, 132, 134–135, and 139–147 (61 residues, or 40.4%, out of the total 167 residues in the protein). RMSDs were calculated between all 25,000 structures generated in the molecular dynamics simulations of the selected homology model, and all 25,000 structures generated in the molecular dynamics simulations of the selected NMR model.

## Results

### CheW Protein Sequences are very Diverse Despite the Conserved Function

**Figure 1:**
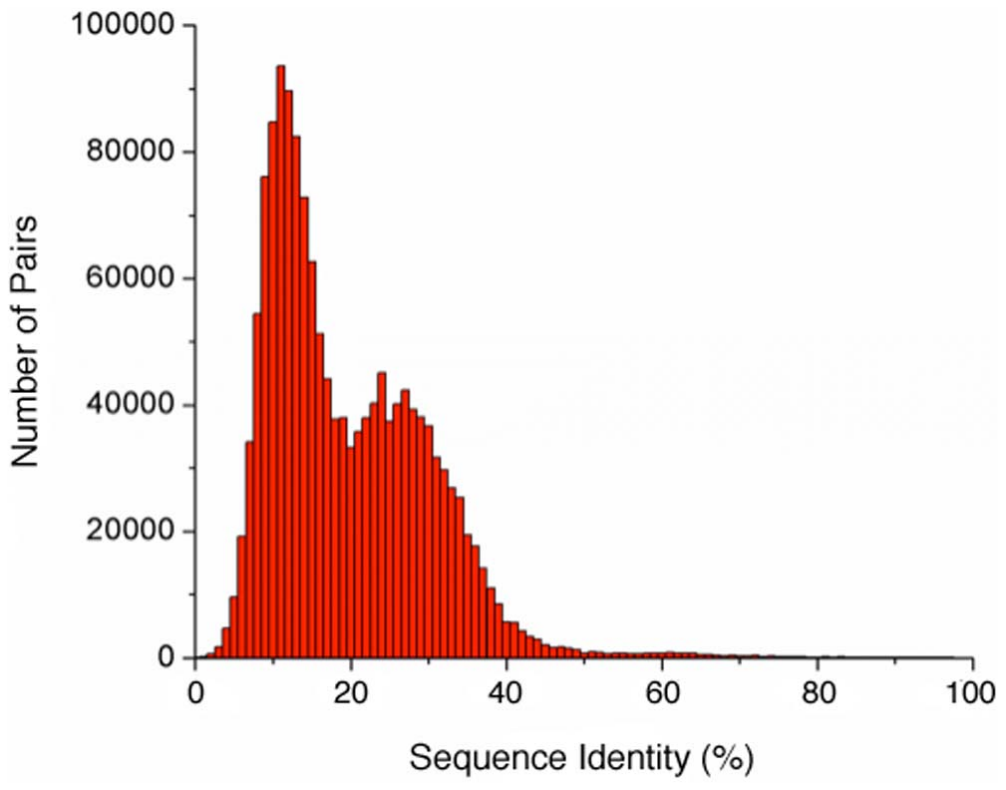
Histogram of the pairwise sequence identities for amongst 1,742 non-redundant CheW sequences. The pairwise identity 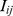 between the sequences *i* and *j* was calculated for 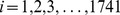 and 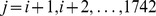. All 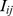 values were binned in 1% bins and displayed in the histogram format.

Pairwise comparisons between 1,742 non-redundant CheW protein sequences from public databases indicate that most sequences exhibit less than 20% identity to each other (Figure 1), thus being in the “Twilight Zone” described above and presenting a challenge for homology modeling. The bimodal distribution observed in Figure 1 is attributed to the presence of three major classes of chemotaxis system: flagellar (F), Type IV pili (Tfp) and alternative cellular function (Acf) [17]. Sequences from flagellar systems are substantially different from Tfp and Acf sequences, and Tfp and Acf are also different from each other. The lowest identity peak shows sequences from two given classes compared to a third class while the peak with higher identity is related to comparisons between sequences from the same classes.

**Figure 2:**
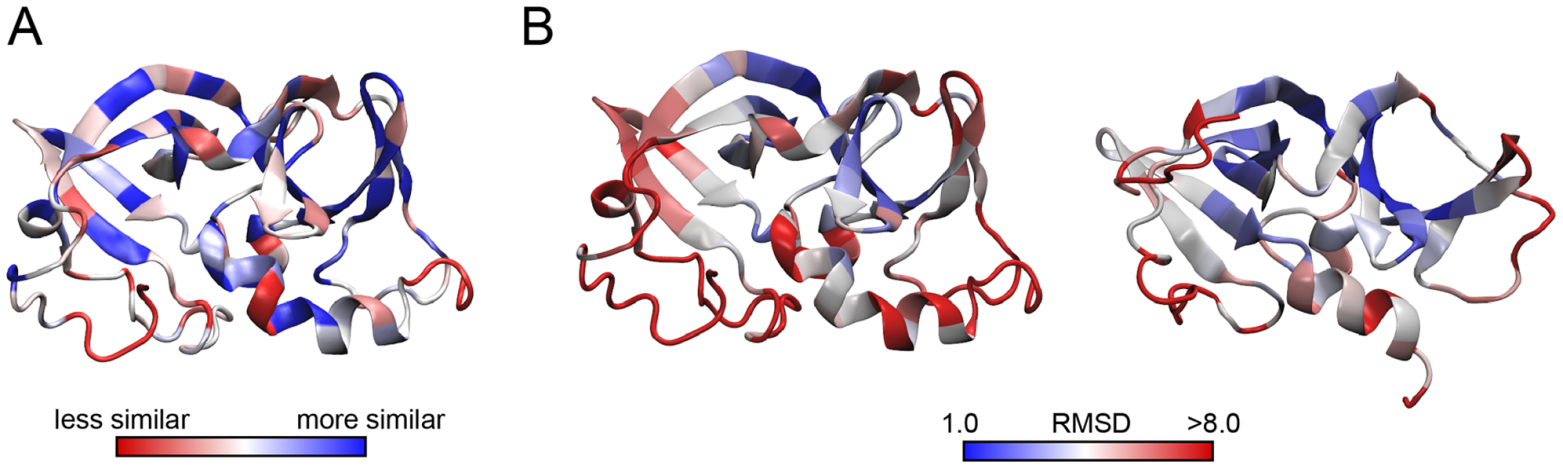
Mapping of sequence and structural similarity into the CheW structure. A. Sequence similarity between *E. coli* CheW and *T. maritima* CheW (BLOSUM60) mapped to *E. coli* NMR structure. B. To measure structure similarity we measure the RMSD per residue between the selected homology model and the NMR structure of *E. coli* (left) and *T. maritima* (right).

Homology models of *E. coli* CheW were built using the NMR structure of *T. maritima* CheW (PDB code: 1K0S) as a template. Similarly, homology models of *T. maritima* CheW were built using the *E. coli* NMR structure as a template (PDB code: 2HO9). The residue identity between the sequences of these two proteins is 25.8%. The sequence similarity score per residue is mapped to the *E. coli* CheW NMR structure (Figure 2.A.). It is a paradigm in molecular evolution that protein regions of biological importance tend to maintain amino acid conservation in a certain position over large evolutionary distances. This does not seem to be clearly the case for the CheW protein. Despite its crucial role as a scaffold protein in chemotaxis, CheW sequences from *T. maritima* and *E. coli* show no spatial co-localization of conserved residues in the 3D structure (Figure 2.A.). This intrinsic characteristic is an additional challenge to make biologically relevant models of CheW proteins.

### Static Homology Models of Individual CheW Proteins are Structurally Different from their Experimental Target

The biological significance of the homology model is analyzed through the calculated RMSD per residue between the top scoring homology model (i.e. the model that would be identified as the “best model” in fully automated homology modeling) and the first structure of the NMR ensemble of each organism (i.e. the experimental structure that would be visualized by most protein structure rendering software). The results are mapped respectively to the *E. coli* and *T. maritima* NMR structures (Figure 2.B.). As a general trend, residues belonging to the hydrophobic core of the protein are accurately modeled despite the low sequence similarity in the region (Figure 2.A.). In contrast, residues further from the center show low positional accuracy, despite being part of secondary structure elements considered as “medium resolution” (atomic RMSDs of 0.57 Å and 1.06 Å for backbone and heavy atoms, respectively for *T. maritima*) and “high resolution” (atomic RMSDs of 0.49 Å and 0.80 Å for backbone and heavy atoms, respectively for the *E. coli* protein) “structured elements” in the NMR structures. This could be due the structural alignment by a least squares fit method used here. However, residues located on the hydrophobic surface formed by the β-strands 1, 2, 3, 6 and 8 (top of the structures in Figure 2) present lower RMSDs per residue than the residues on the α-helix 1 and 2 and surrounding structural elements (bottom of the structures in Figure 2). Interestingly, multiple experiments suggest that same hydrophobic surface to be involved in MCP binding (Table S1 in File S1 and Figure S3 in File S1). On the other hand, the region with residues interacting with the kinase, formed by β3–β4 loop and β-strands 4 and 5 (right side of the structures in Figure 2), shows a large disparity between the NMR structure and the homology model in CheW proteins from both organisms.

### Ensembles of Static CheW Homology Models also Exhibit Structural Differences with their Experimental Targets

**Figure 3:**
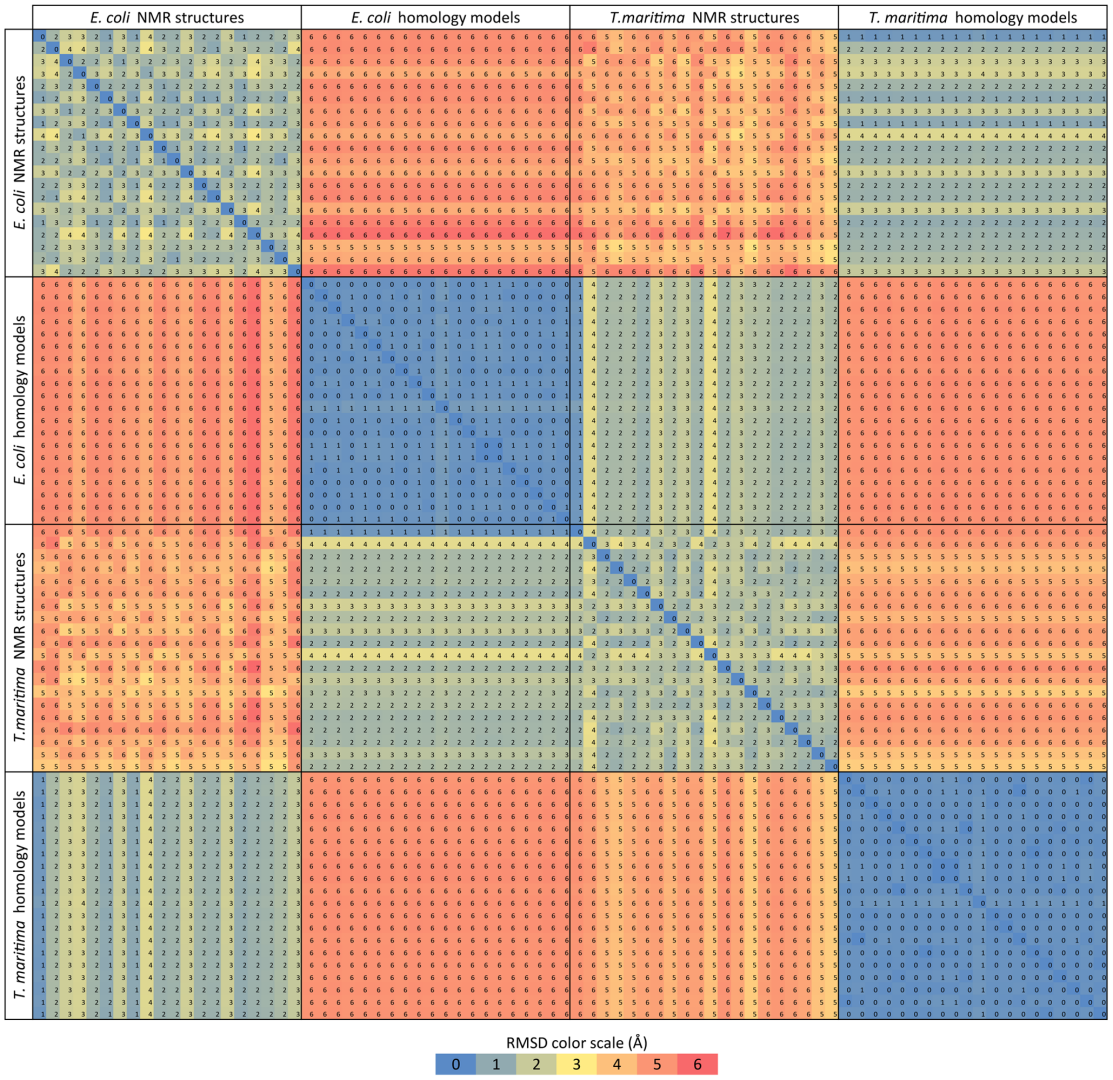
Comparison of the RMSDs between 20 homology models and 20 NMR structures. Prior to the RMSD calculation of each pair, the structures were aligned, taking into consideration the backbone atoms of the residues that can be aligned without gap in the protein cheW from *E. coli* and *T. maritima* pairwise alignment. The selected residues for *E. coli* are: 7 to 72, 74 to 120, 123 to 151, and 154 to 161, while for *T. maritima*, all residues were included except 151. The RMSD values calculated for the same set of residues used in the alignment were calculated using the measure RMSD function of VMD.

**Figure 4:**
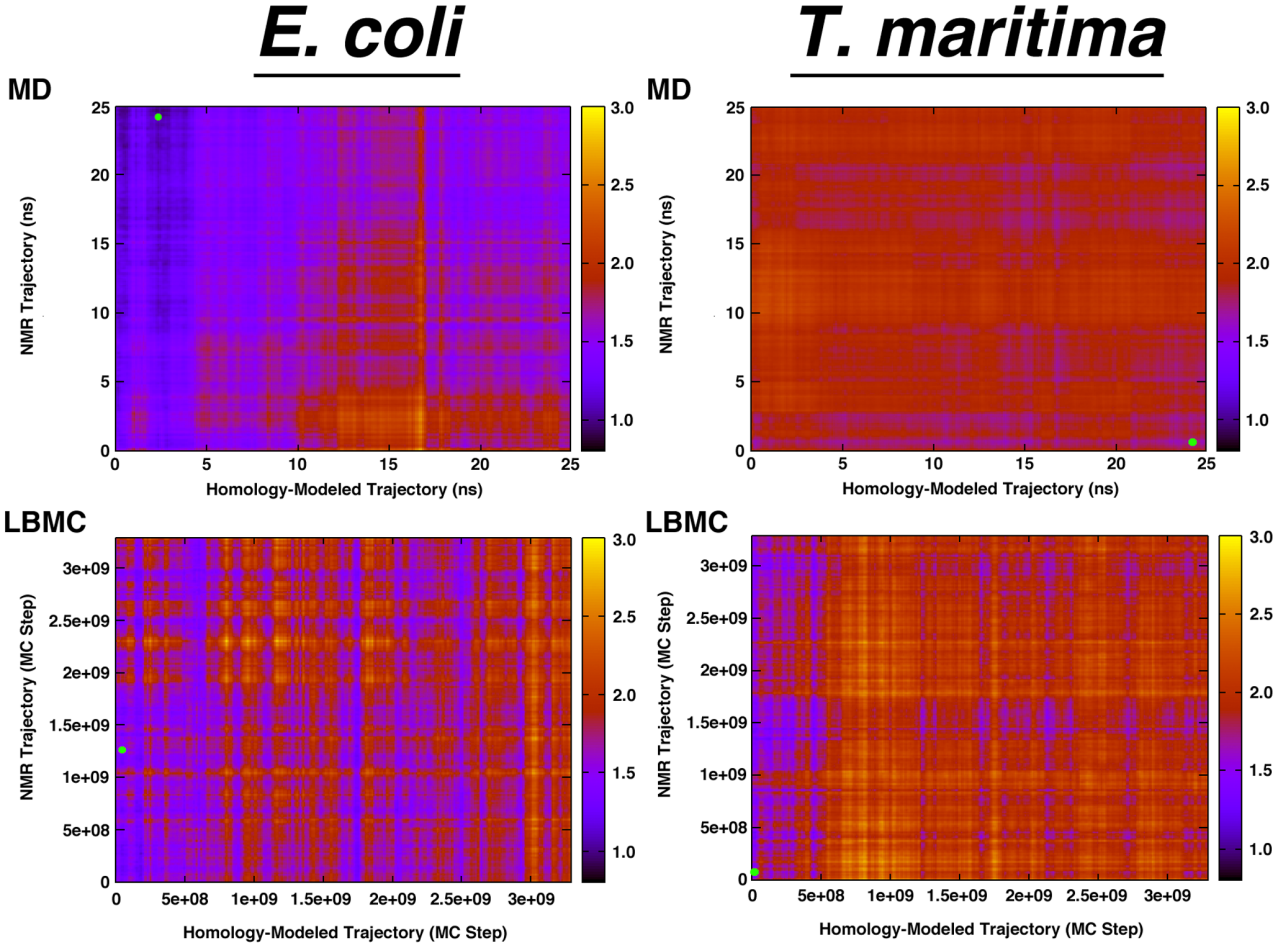
RMSD Similarity Matrices. RMSD matrices comparing the similarity of each point of the homology-modeled trajectories with each point of the NMR trajectories for the α/β consensus regions. The top two matrices are for the MD simulations, and the bottom two are for the LBMC simulations. The small green dots in each graph indicate the lowest RMSD values.

The homology models are structurally closer to their template structure than to their target structures. Comparison of the RMSDs calculated between the ensemble of 20 homology models and the ensemble of 20 NMR structures (Figure 3) exhibit relatively large values, up to 6.5 Å for *E. coli* and up to 6 Å for *T. maritima* proteins, suggesting that in the case of *E. coli* homology models, CheW is further away from the NMR model than in the case of *T. maritima.* In contrast, the RMSD values between the experimental static NMR sub-structures are no larger than 4 Å for both *E. coli* and *T. maritima* proteins. Molecular dynamics and Monte Carlo simulations of specific homology models and NMR structures (see Materials and Methods) sample structural variations that are thermodynamically accessible at room temperature. RMSD values between the 50,000 structures obtained by MD simulation based on NMR and homology models were calculated as described in the Materials and Methods section. The results are summarized in Tables 1 & 2, and, in the case of the α/β consensus residues, displayed on Figure 4. The “all residue” RMSD values are high: 9.1 Å for *E. coli* and 6.8 Å for *T. maritima* (Tables 1 & 2). However, for “residues 17–157” (core structural elements of the proteins), RMSD values are lower and they are further lowered for the α/β consensus residues ranging between 3.0 Å for *E. coli* and 4.4 Å for *T. maritima* proteins. This indicates that about half to two-thirds of the relatively high overall RMSD difference between the homology model and NMR structures is mostly due to differences in the terminal flexible regions of the protein, with the structural core being better modeled by the homology approach.

**Table 1:** RMSD values (Å) for MD simulation trajectories, from Figure 4.

RMSD vs. NMR	*E. coli* CheWAll Residues	E. coli CheWRes. 17–157Only	*E. coli* CheWα/β Consensus Residues Only	*T. maritima* CheWAll Residues	*T. maritima* CheWResidues10–147 Only	*T. maritima* CheWα/β Consensus Residues Only
Starting value	9.1	6.2	3.0	6.8	5.8	4.4
Lowest value	5.2	3.0	0.8	3.4	2.8	1.5
Highest value	10.4	6.3	3.4	6.0	5.1	2.7
Average value	8.5	5.3	2.1	5.3	4.5	2.4

**Table 2:** RMSD values (Å) for LBMC simulation trajectories, from Figure 4.

RMSD vs. NMR	*E. coli* CheWAll Residues	E. coli CheWRes. 17–157Only	*E. coli* CheWα/β Consensus Residues Only	*T. maritima* CheWAll Residues	*T. maritima* CheWResidues10–147 Only	*T. maritima* CheWα/β Consensus Residues Only
Starting value	9.1	6.2	3.0	6.8	5.8	4.4
Lowest value	4.9	3.9	1.1	3.9	3.5	1.1
Highest value	11.4	6.8	2.9	9.1	6.8	6.3
Average value	8.1	5.2	1.9	6.0	4.9	3.4

### Conformational Sampling of the CheW Homology Models Reduces their Difference with Experimental Targets

**Figure 5:**
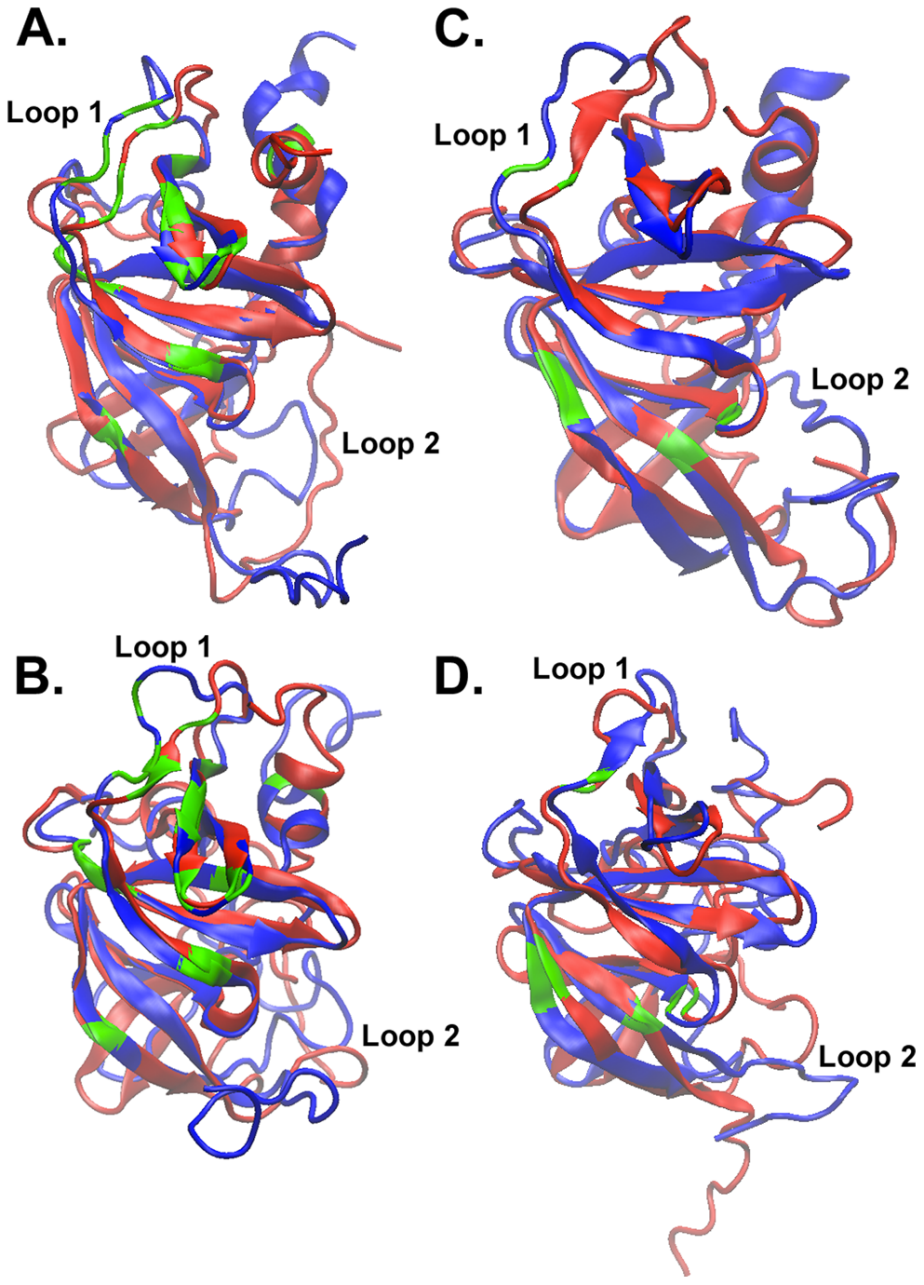
Structural Superimposition of Similar Models. Superimposition of the most similar structure in the NMR trajectory (red) with the most similar structure in the homology-modeled trajectory (blue) for (A): *E. coli* MD simulation (RMSD = 0.8 Å), (B) *E. coli* LBMC simulation (RMSD = 1.0 Å), (C) *T. maritima* MD simulation (RMSD = 1.5 Å), and (D) *T. maritima* LBMC simulation (RMSD = 1.1 Å). The ribbon segments colored in green indicate the residues that are proposed to participate in protein-protein interactions (c.f. Supplementary Information: Table S1 in File S1).

Molecular dynamics and Monte Carlo trajectories sample structural variations of the starting NMR and homology modeled structures that can be much closer to each other than the static starting models are to each other: as low as 0.8 Å (for *E. coli*) and 1.5 Å (for *T. maritima*) for the α/β consensus residues (Table 1). Superimposition of the corresponding structures shows that the core structures are highly similar in NMR and homology modeled trajectories (Figure 5). Yet there is very little overlap seen in the N-terminal and C-terminal regions as well as in some of the internal loops. Significant differences are also visible in the β3–β4 loop (Loop 1) located near the top of each structure in Figure 5 (corresponding to residues 42–56 in *E. coli* and residues 37–49 in *T. maritima* proteins).

**Figure 6:**
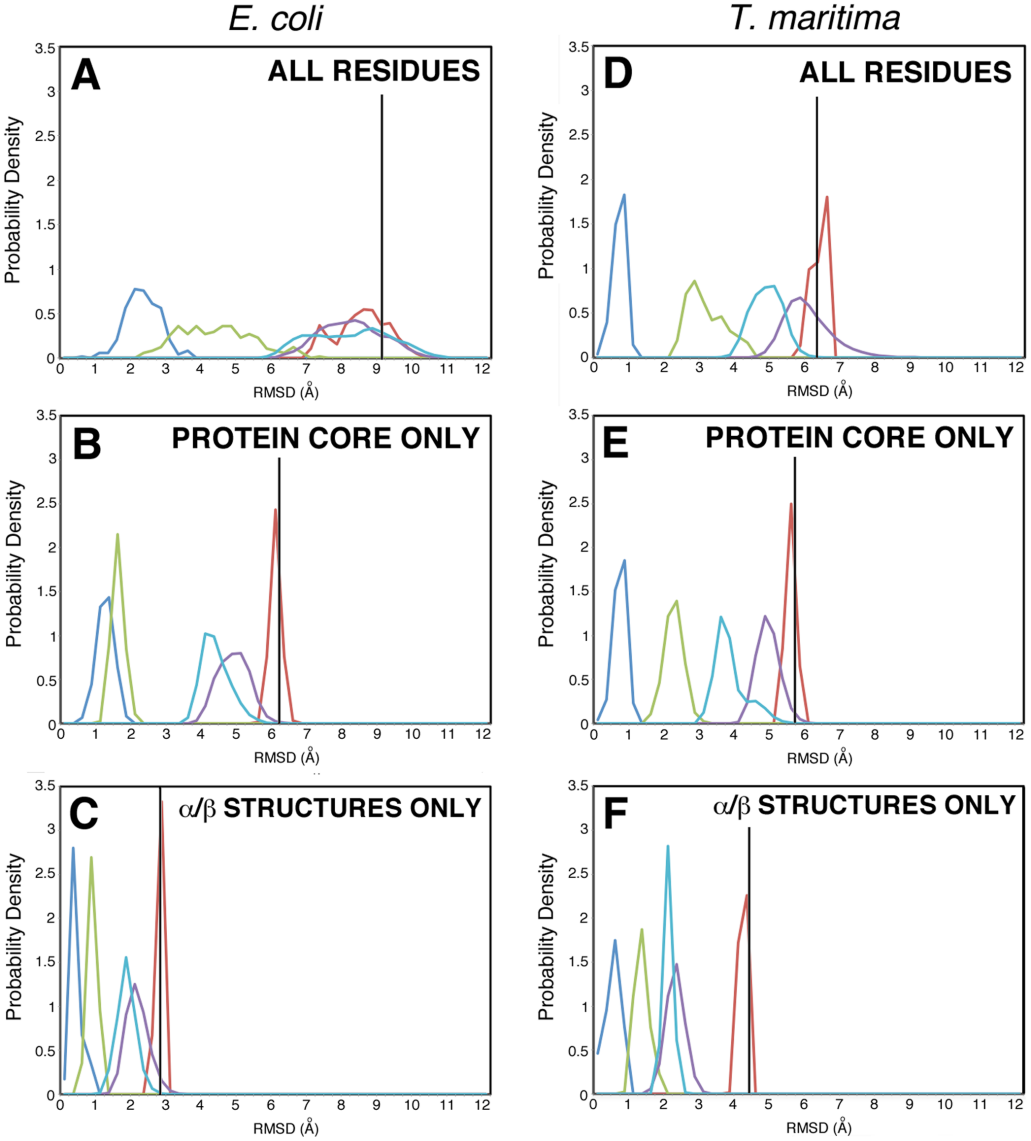
Histograms of the RMSD values comparing the NMR ensembles and MD/LBMC simulated trajectories. Blue: RMSD values of the 20 homology models versus each other; Green: RMSD values of the 20 NMR structures versus each other; Red: RMSD values of the 20 homology models versus 20 NMR structures; Purple: RMSD values of every structure of the homology model simulation versus every structure of the NMR simulation, using LBMC; Cyan: RMSD values of every structure of the homology model simulation versus every structure of the NMR simulation, using MD. The vertical black line indicates the starting RMSD value between the homology model and the NMR structures simulated by MD or LBMC.

Figure 6 shows the distributions of RMSD values between the structures in the NMR and homology model simulations (as shown in Figure 2 for the α/β consensus residues). In all of the cases represented in Figure 6, the distribution representing the 20 homology models vs. each other (blue) consistently exhibit the lowest range of RMSD values, indicating that they are relatively structurally close to each other. The RMSD range of the 20 NMR structures vs. each other (green) consistently exhibit RMSD values shifted toward higher values than the homology models, indicating that the NMR structures collectively describe more configurational space than the homology models. This configurational space is different when calculating RMSDs between the homology models and the NMR structures (red). As shown in Supplementary Information (Table S1 in File S1) and described above, the NMR and homology models are closer to each other in the case of the core residues than when comparing all residues, and further so in the case of the α/β consensus residues. The purple and cyan distributions show that the range of conformations sampled in molecular dynamics and LBMC simulations are slightly different in the case of simulations of the selected NMR model and of the selected homology model. Importantly, the red distribution in Figure 6.E. shows that, in the case of the MD simulation of the *E. coli* homology model and of the *E. coli* NMR structure, the configurational space sampled is leading to RMSD values that can be lower than that exhibited between NMR structures. In other words, a static NMR model and a static homology model, differing by approximately 3 Å RMSD (black line in Figure 6.E.) can, when sampling their accessible configurational space, find themselves closer to each other at RMSD values less than 1 Å than individual NMR structures of the same protein.

## Discussion

### Quality Homology Models of CheW can be Successfully Constructed using Templates of Low Sequence Identities

When comparing CheW homology models to their corresponding experimental structures, the limitation of homology modeling becomes apparent: models are structurally closer to their template structure than to their target structures. CheW is known to have two distinct interacting surfaces that are equally important. Mutations in residues in either of these surfaces disrupt chemotaxis [44], [45]. In the present study, the MCP binding site is better modeled than the CheA binding site. This difference is likely due to the β3–β4 loop being part of the interacting surface with the kinase, while the MCP binding site consists of well-defined beta strands. Overall, the regions of the structures corresponding to the structural core exhibit more conserved sequences (30 to 35% identity) than the regions outside of the structural core (10 to 15% identity), indicating that structural conservation is correlated to sequence conservation for CheW and that the sequence conservation varies in different parts of the protein. However, sampling of the local folding landscape is needed to translate this higher sequence identity into better structural predictions for CheW. Although homology models and NMR models of CheW may be overall different from each other, the sampling of structural space accessible by these models using molecular dynamics or Monte Carlo simulations significantly improves the agreement between predicted and experimental models of the same protein. Predicted structures may be closer to each other than NMR structures are close to each other. This suggests that whenever possible, individual, static homology models should not be seen as “the best possible model”, but rather as a possible model amongst an ensemble. A homology model (or, better, an ensemble of homology models) should be subjected to MD or Monte Carlo simulations to identify the range of thermodynamically accessible structures. Relatively short molecular dynamics simulations such as the ones presented here are beneficial: the lowest RMSD between the NMR-simulated trajectory and the homology-modeled trajectory (green dot in Figure 4), is typically near the end of one simulation’s configurational space and the beginning of the other, which indicates that conformational changes happen beyond the local rearrangements of the first stages of molecular dynamics simulations (LBMC graphs in Figure 4 do not indicate time-dependent properties). In the present simulations, different starting conformations explore regions of the conformational space that approaches the configurational space of each other. However, comparisons between homology models and very long molecular dynamics simulations of experimental structures of proteins and of corresponding homology models indicated that the homology model can become quite different from their experimental targets, most likely because of limitations in the accuracy of the force field [46]. It is possible that the improvement of the homology models’ quality due to MD is limited to relatively short simulation times limiting the sampling to the local accessible space.

### Quality CheW Homology Models Require Caution when Functionally Interpreted

While experimentally determined structures are currently available only for three CheW proteins, experimental studies on chemotaxis are carried out in dozens of bacterial species [16]. Furthermore, static models are generated for remote CheW homologs (as distant as spirochetes) using *E. coli* and *T. maritima* templates in order to draw conclusions on their structural similarities [19] and static models of CheW and its interacting partners are produced in order to obtain a higher-order assembly of the chemotaxis signaling complex [10], [11]. *T. maritima* is not genetically tractable and structural information obtained for its chemotaxis proteins using crystallography and NMR must be translated onto homologs in other species, where predicted interactions can be verified using genetics and biochemistry. These recent developments in the field of bacterial chemotaxis necessitate better understanding of usefulness and limitations of homology models built on templates with low sequence similarity.

Molecular dynamics and MC simulations still fail to correctly predict and explore the structure of the highly flexible N-terminal and C-terminal regions of the CheW homology models. This is also the case for the flexible β3–β4 and β8–β9 loops (Loop 1 and Loop 2). What does this mean in terms of confidence of the model when it comes to translating CheW *structure* into *function*? Supplementary Information (Figure S3 in File S1 and Table S1 in File S1), show the CheW residues that have been proposed to interact with chemoreceptors and the CheA histidine kinase, based on experimental evidence from mutagenesis, protein footprinting, and NMR studies [47], [48], [49], [50]. These residues are also highlighted in green in Figure 5, many of them being located in the stable protein core. Some residues of interest are also in the flexible Loop 1 as well, suggesting that protein dynamics may also play a role in the function of CheW. The α/β consensus region, for which the “dynamics-improved” homology models are in agreement with the structures in the NMR ensemble, contains many residues that are proposed contact sites for chemoreceptor binding. However, this is not necessarily the case for some residues proposed to participate in interactions between CheW and CheA kinase located in the structurally variable regions that are not well predicted by homology modeling. Specifically, the dynamic β3–β4 loop between residues 42–56 in *E. coli* and residues 37–49 in *T. maritima,* which is difficult to model correctly, is the known site for the interaction between CheW and the CheA P5 domain, suggesting that molecular dynamics and other methods of assessing protein flexibility will play a key role in the study of this chemotaxis complex.

### Conclusion

This work shows that it is possible to construct a reliable ensemble of CheW homology models despite low sequence identity between a CheW target sequence and its template. A key component of this modeling should consist of an adequate sampling of configurational space using molecular dynamics or Monte Carlo simulations. Homology models of CheW should be viewed as snapshots of an otherwise large ensemble of accessible conformational space. This ensemble suggests that most of the potential predicted CheW/MCP interactions are overall correctly modeled, but that the potential interactions between CheW and CheA involved regions that are more difficult to model and thus are less reliable. Revealing the dynamics of predictive homology models of CheW will aid the assembling of the chemotaxis complex and understanding the mechanism of signal transduction. Assembling the chemotaxis complex of *E. coli*, for which the wealth of genetic, biochemical and imaging data has been accumulated, will require modeling of the CheA kinase, the central regulator of chemotaxis. No structure is currently available for the *E. coli* CheA protein and current [11] and future efforts are likely to use the *T. maritima* CheA templates for homology modeling. CheA is a much larger and more complex protein than CheW. Therefore, potential problems with modeling revealed here will only multiply. Consequently, longer molecular dynamics simulations will be needed to aid in this important task.

## Supporting Information

File S1 **Figures S1, S2, S3 and Table S1.**
(DOCX)
