# Supplementary material for: Homology Modeling of the CheW Coupling Protein of the Chemotaxis Signaling Complex
Source: PLoS One. 2013 Aug 7;8(8):e70705. doi: 10.1371/journal.pone.0070705 (PMC3737408; doi:10.1371/journal.pone.0070705)
Supplement: File S1 — Figures S1, S2, S3 and Table S1. (DOCX) [file pone.0070705.s001.docx]

**Cashman et al. Homology Modeling of the CheW Coupling Protein of the Chemotaxis Signaling Complex**

**Supporting Information File S1**

**Figures S1-S3**

**Table S1**

I. Comparison of Sequence Alignments Between CheW From *E. coli* and *T. maritima*.


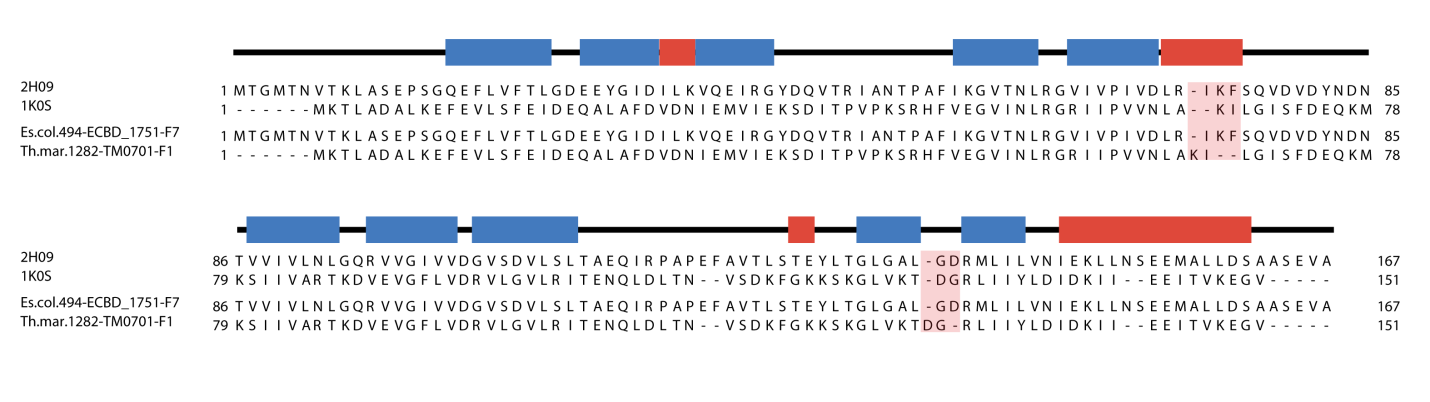


**Figure S1.** Comparison of sequence alignments between CheW homologs from *E. coli* and *T. maritima* built with two different methods. First 2 sequences: from a direct alignment of *E. coli* vs. *T. maritima* using the program MOE as described in Materials and Methods, subsection *Homology Modeling*. Third and fourth sequences: aligned as follow: All 3,738 CheW proteins sequences from draft and complete genomes were collected using the August 2012 release of the MIST database [[1](#_ENREF_1)]. Using HMM provided by the authors [[2](#_ENREF_2)] and HMMER 2.3.2 [[3](#_ENREF_3)] the CheW sequences were assigned to classes [[2](#_ENREF_2)]. The sequences from classes F1 (*T. maritima*) and F7 (*E. coli*) were selected and separated according to their chemotaxis classes in individual files. Each file was subjected to multiple sequence alignment using algorithm L-INS-I from the package MAFFT [[4](#_ENREF_4)]. The two multiple sequence alignment were then aligned by profile alignment from MAFFT package. The sequences from *E. coli* and *T. maritima* were selected from the profile alignment and manually inspected.

The alignment differences between the MOE-based and the MAFFT-based approaches are highlighted in red. Secondary structure elements in *E. coli* structure 2HO9 are shown in in blue (beta strand) and red (alpha helices).

**References for Figure S1:**

1. Ulrich LE, Zhulin IB (2010) The MiST2 database: a comprehensive genomics resource on microbial signal transduction. Nucleic Acids Res 38: D401-407.

2. Wuichet K, Zhulin IB (2010) Origins and diversification of a complex signal transduction system in prokaryotes. Sci Signal 3: ra50.

3. Eddy SR (1998) Profile hidden Markov models. Bioinformatics 14: 755-763.

4. Katoh K, Toh H (2008) Recent developments in the MAFFT multiple sequence alignment program. Brief Bioinform 9: 286-298.

II. RMSD vs. Time/Frame for Simulated Trajectories.

**
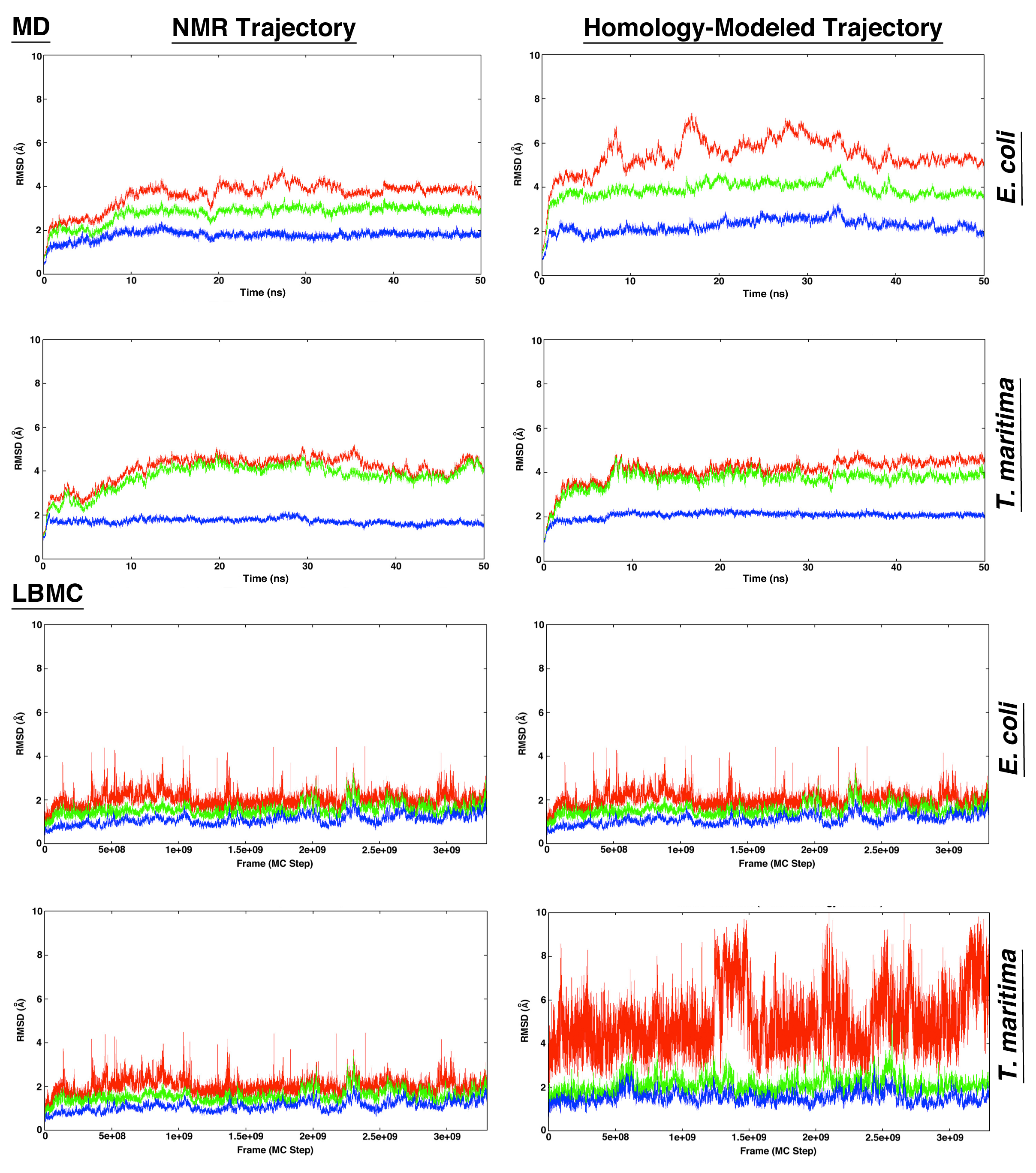
**

**Figure S2.** RMSD vs. Time for the molecular dynamics and RMSD vs. Frame for the LBMC simulations of CheW for *E. coli* and *T. maritima*. Red lines indicate the RMSD of the complete protein (all residues), green lines indicate the RMSD of the protein core only (residues 17-157 for E. coli and residues 10-147 for T. maritima), and blue lines indicate the RMSD of the α/β consensus residues only.

III. Experimental Evidence of Protein Interaction Sites With CheA and MCP Receptor.

**
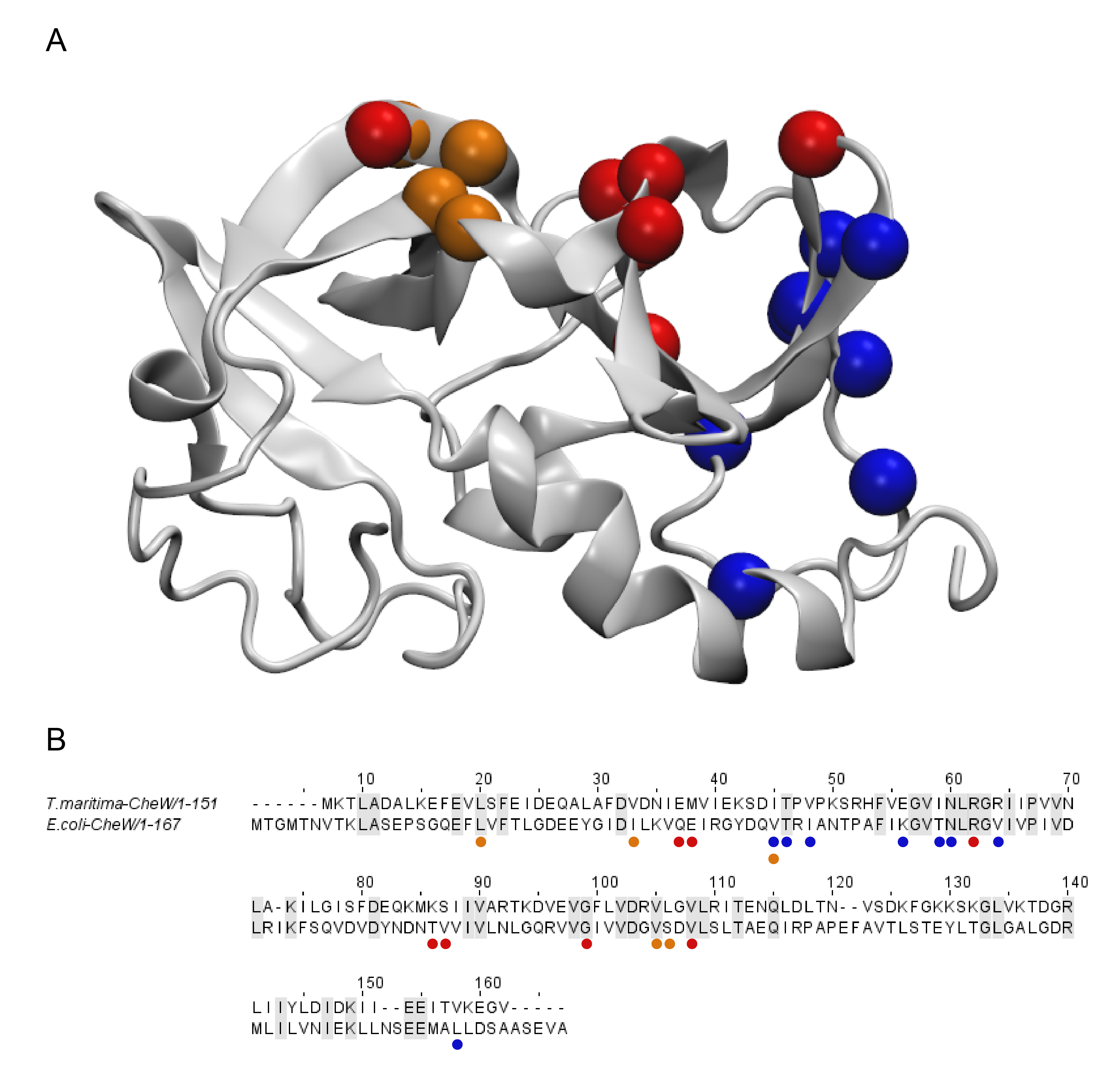
**

**Figure S3.** A. Experimental evidence of protein interaction sites of CheW with the CheA histidine kinase (blue) and the methyl-accepting chemotaxis (MCP) receptor (red for data generated in studies in *E. coli* and orange for studies in *T. maritima*) mapped to the *E. coli* CheW NMR structure. The data is summarized in Table 1. Interacting residues from studies in *T. maritima* were mapped to the *E. coli* structure using the pairwise alignment between the two sequences. B. Pairwise alignment between *E. coli* and *T. maritima* sequences. Circles are colored according to panel A and grey shade emphasizes identical residues.

**TABLE S1.** CheW residues proposed to participate in protein-protein interactions.

| **Protein Partner** | **Organism** | **Residues** | **Reference** |
| --- | --- | --- | --- |
| Chemoreceptor | *T. maritima* | 14 27 39 98 99 | [1] |
| Chemoreceptor | *E. coli* | 37 62 | [2] |
| Chemoreceptor | *E. coli* | 38 87 | [3] |
| Chemoreceptor | *E. coli* | 38 62 99 88 86 108 | [4] |
| Kinase | *E. coli* | 46 48 59 60 64 | [2] |
| Kinase | *E. coli* | 45 46 56 158 | [3] |

**References for Table S1:**

1. Vu, A., Wang, X., Zhou, H., and Dahlquist, F. W. (2012) The receptor-CheW binding interface in bacterial chemotaxis, *J. Mol. Biol.* *415*, 759-767.

2. Underbakke, E. S., Zhu, Y., and Kiessling, L. L. (2011) Protein footprinting in a complex milieu: identifying the interaction surfaces of the chemotaxis adaptor protein CheW, *J. Mol. Biol.* *409*, 483-495.

3. Boukhvalova, M., VanBruggen, R., and Stewart, R. C. (2002) CheA kinase and chemoreceptor interaction surfaces on CheW, *J. Biol. Chem.* *277*, 23596-23603.

4. Liu, J. D., and Parkinson, J. S. (1991) Genetic evidence for interaction between the CheW and Tsr proteins during chemoreceptor signaling by *Escherichia coli*, *J. Bacteriol.* *173*, 4941-4951.
